# Discrete Shearlets as a Sparsifying Transform in Low-Rank Plus Sparse Decomposition for Undersampled (*k*, *t*)-Space MR Data

**DOI:** 10.3390/jimaging8020029

**Published:** 2022-01-29

**Authors:** Nicholas E. Protonotarios, Evangelia Tzampazidou, George A. Kastis, Nikolaos Dikaios

**Affiliations:** 1 Department of Applied Mathematics and Theoretical Physics, University of Cambridge, Cambridge CB3 0WA, UK; 2 Mathematics Research Center, Academy of Athens, 11527 Athens, Greece; evitzam@phys.uoa.gr (E.T.); gkastis@academyofathens.gr (G.A.K.); ndikaios@academyofathens.gr (N.D.); 3 Department of Physics, National Kapodistrian University of Athens, 15771 Athens, Greece

**Keywords:** MRI, image reconstruction, robust principal component analysis (RPCA), discrete shearlet transform, low-rank plus sparse decomposition, dynamic contrast enhanced (DCE), small bowel imaging

## Abstract

The discrete shearlet transformation accurately represents the discontinuities and edges occurring in magnetic resonance imaging, providing an excellent option of a sparsifying transform. In the present paper, we examine the use of discrete shearlets over other sparsifying transforms in a low-rank plus sparse decomposition problem, denoted by L+S. The proposed algorithm is evaluated on simulated dynamic contrast enhanced (DCE) and small bowel data. For the small bowel, eight subjects were scanned; the sequence was run first on breath-holding and subsequently on free-breathing, without changing the anatomical position of the subject. The reconstruction performance of the proposed algorithm was evaluated against *k*-*t* FOCUSS. L+S decomposition, using discrete shearlets as sparsifying transforms, successfully separated the low-rank (background and periodic motion) from the sparse component (enhancement or bowel motility) for both DCE and small bowel data. Motion estimated from low-rank of DCE data is closer to ground truth deformations than motion estimated from *L* and *S*. Motility metrics derived from the *S* component of free-breathing data were not significantly different from the ones from breath-holding data up to four-fold undersampling, indicating that bowel (rapid/random) motility is isolated in *S*. Our work strongly supports the use of discrete shearlets as a sparsifying transform in a L+S decomposition for undersampled MR data.

## 1. Introduction

Robust principal component analysis (RPCA) techniques [1,2] have been proposed to decompose dynamic MR images to a low-rank and a sparse component. The low-rank should ideally contain the background and periodic motion, whereas the sparse component should include rapid intensity changes including noise, signal enhancement, non-periodic deformations, etc. Reconstruction algorithms from undersampled data based on RPCA have been previously suggested in the literature [3,4,5,6], and were applied mainly to dynamic contrast enhanced (DCE) and cardiac perfusion data [7]. Previous studies involving RPCA-related medical imaging analysis were performed in order to isolate (i) noise from the signal [6], (ii) enhancement in DCE imaging from the background respiratory motion [4,5,8], and (iii) bowel motility from respiratory motion [9]. A potential benefit in DCE image registration involves the fact that motion will be estimated from the low-rank, where intensity changes due to enhancement are not present, leading to more accurate image registration [8]. Furthermore, RPCA is well-poised for small bowel data, since breathing is periodic (low-rank) and bowel motility is comparatively unperiodic (sparse), and the estimated bowel motility from the sparse component, isolated from respiratory motion, has the potential to serve as a useful biomarker in a range of gastrointestinal disorders [8]. Small bowel motility has already been shown to correlate with inflammatory activity [10,11]. If the signal is composed from a low-rank and a sparse component, RPCA has been proven to perform better on reconstruction from undersampled data than compressed sensing (CS) [12,13] and joint low-rank and sparsity constraint methods [6]. Increasing temporal resolution is crucial both in DCE imaging, for a better depiction of the pharmacokinetics, and in small bowel imaging, in order to allow for the observation of high frequency contractions. Alternatively, the aim of undersampling could be volumetric acquisitions that require longer scan times. Volumetric acquisitions may prove helpful for the improvement of physiological coherence [14].

This paper postulates that the use of discrete shearlets (DS) as a sparsifying transform can benefit RPCA decomposition/reconstruction. Choosing the appropriate sparsifying transform where our signal is sparse is expected to improve the reconstruction performance, especially for high undersampling factors. Discrete shearlets were introduced by Guo et al. [15] in order to accelerate and optimize the reconstruction process by applying shear transformations in various directions in two-dimensional objects with edges. Based on [15], Yi et al. [16] employed relevant algorithms for extracting and detecting details about edges, including junctions with added noise on the image. Furthermore, the numerical implementation applied by Hauser et al. in [17] established the shearlet transformation for several scales and directions. Recently, Yuan et al. [18] proposed a new reconstruction algorithm, utilizing the non-subsampled shearlet transform (NSST) sparsity prior in undersampled data for brain MR images, to promote sparser representations and higher directional sensitivity, thus eliminating artifacts and reconstructed errors. In addition, the shearlet transform has been employed in several recent studies, including the hybrid regularization method for CS MRI incorporating total variation [19], the CS algorithm for fast 3D cardiac MR imaging using iterative reweighting [20], and the shearlet-based CS technique combined with nonlocal total variation [21].

Our hypothesis is that discrete shearlets are the optimum sparsifying transform to represent edges in multi-dimensional data, such as DCE and small bowel imaging. To evaluate our hypothesis, we have compared different sparsifying transforms, including no transform, temporal Fourier transform, and discrete shearlets, in terms of decomposition/reconstruction performance. The developed RPCA reconstruction algorithm is evaluated (i) on simulated DCE data, and (ii) on eight different small bowel MR datasets, acquired during breath-holding and free-breathing modes (one after the other in a single scan). The reconstruction performance of the suggested RPCA reconstruction algorithm, using different sparsifying transforms, is compared to a widely used focal underdetermined system solver, namely, *k*-*t* FOCUSS [22]. In terms of decomposition, it is examined whether deriving deformations (i) from the low-rank of DCE data will be similar to the ground truth respiratory motion, and (ii) from the sparse component of the free-breathing data will be similar to the deformations from the sparse component of the breath-holding data, where less respiratory motion is assumed.

## 2. Theory

### 2.1. Focal Underdetermined System Solver (k-t FOCUSS)

Tsao et al. [23] suggested the broad-use linear acquisition speed-up technique, known as *k*-*t* BLAST, that employs a training low-resolution dataset to obtain signal correlations; then, *k*-*t* BLAST utilizes these signal correlations as prior information in order to recover spatio-frequency (*x*-*f*) images ρ without aliasing artifacts, namely:

(1) $$\rho =\rho_{0}+CF_{t}^{H}{\left(F_{t}CF_{t}^{H}+\lambda I\right)}^{-1}\left(y_{alias}-F_{t}\rho_{0}\right),$$

where $\rho_{0}$ is a complex value baseline image, $C=W\cdot W^{H}$ is the covariance matrix of the signal deviation from the baseline $\rho_{0}$, superscript *H* denotes Hermitian transpose, *W* is a weighting matrix, $F_{t}$ is the Fourier transform along the spatio-frequency direction, λ is a Lagrangian multiplier, and $y_{alias}$ is the aliased signal, initially denoted by *x*, from the low resolution dataset, i.e.,

(2) $$y_{alias}=F_{t}x.$$

Gorodnitsky et al. [24] suggested a novel sparse reconstruction method based on *k*-*t* BLAST, called “focal underdetermined system solver” (*k*-*t* FOCUSS) [25]. *k*-*t* BLAST is considered the first iteration of *k*-*t* FOCUSS. The main novelty of *k*-*t* FOCUSS entails the fact that it updates the weighting matrix *W* iteratively. Jung et al. [22,25] modified *k*-*t* FOCUSS and made it suitable for dynamic MRI applications.

### 2.2. Robust Principal Component Analysis (RPCA)

The low-rank and sparse constraints can be applied simultaneously to promote sparsity and time coherence (such as in *k*-*t* SLR [26]), i.e.,

(3) $$\min_{M}\frac{1}{2}{∥F_{u}M-y∥}_{2}^{2}+\lambda_{L}{∥M∥}_{*}+\lambda_{S}{∥\Phi M∥}_{1},$$

where *M* is the recovered image, considered as an approximately low-rank Casorati matrix, with spatio-temporal dimensions denoted by $M_{x}$, $M_{y}$, $M_{t}$; $F_{u}$ denotes the undersampled Fourier transform, ${∥\cdot ∥}_{2}$ is the $L^{2}$-norm, ${∥\cdot ∥}_{1}$ is the $L^{1}$-norm, ${\left||\cdot |\right|}_{*}$ is the nuclear norm, Φ(·) is a sparsifying transform, and $\lambda_{L}$, $\lambda_{S}$ are trade-off parameters between the consistency term, and the nuclear-norm and $L^{1}$-norm, respectively. The choice of sparsifying transform Φ is crucial to ensure *S* is not low-rank and that ${F_{u}}^{T}y$ is incoherent to *S*.

The idea behind RPCA algorithms is to apply low-rank and sparsity constraints “sequentially”, aiming to decompose the low-rank *L* from the sparse component *S* of the reconstructed image *M*. By separating the background (*L*) from the sparse component (*S*), *S* will become sparser than M=L+S; this will benefit the reconstructions compared to other schemes that use solely or simultaneously the low-rank and sparse constraints [12,26]. RPCA reconstruction (L+S) can be performed via the following unconstrained optimization problem:

(4) $$\min_{L,S}\frac{1}{2}{∥F_{u}\left(L+S\right)-y∥}_{2}^{2}+\lambda_{L}{∥L∥}_{*}+\lambda_{S}{∥\Phi S∥}_{1}.$$

**Figure 1:**
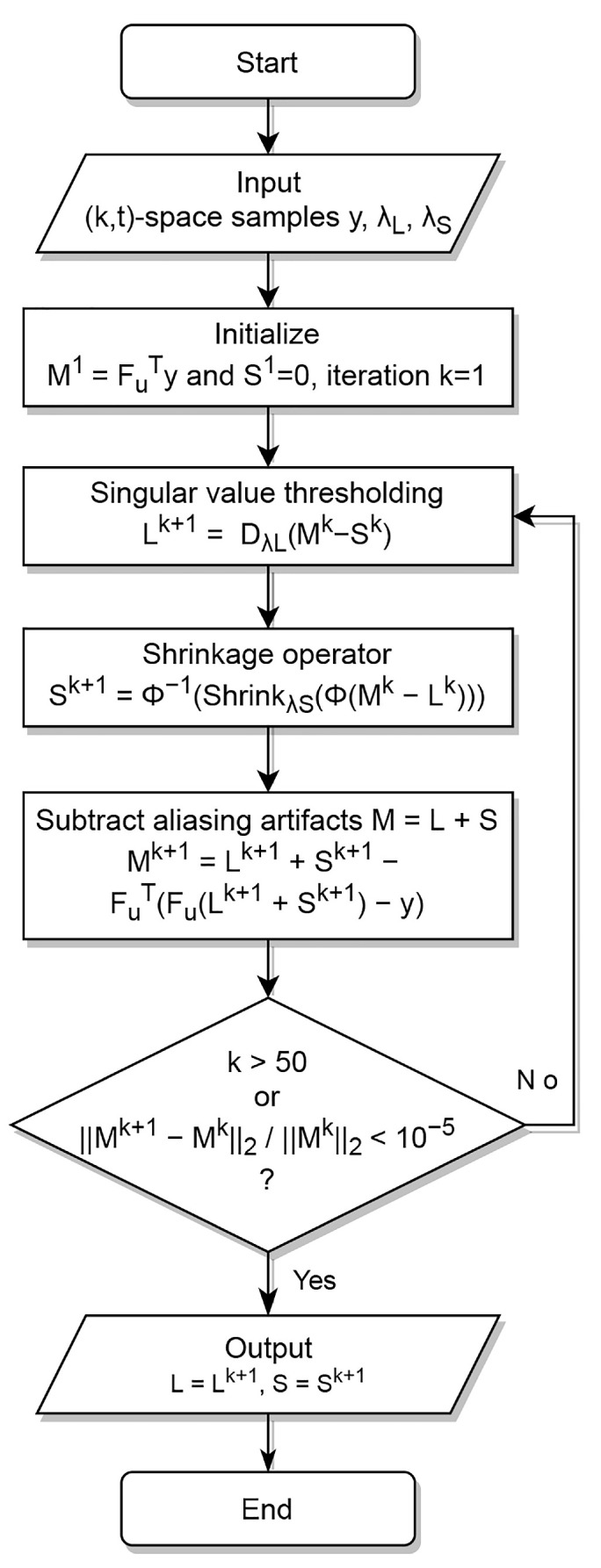
Flowchart of L+S decomposition and reconstruction algorithm, as in [6].

Equation (4) can be solved using split Bregman alternating direction methods [3,4,5,27]. To achieve separation between *L* and *S*, they need to be incoherent, whereas to reconstruct unbiased images from undersampled data, ${F_{u}}^{T}y$ needs to be incoherent with *L* and *S*. The RPCA decomposition implemented in this work was the one suggested by Otazo et al. [6], where singular value thresholding for the low-rank, and a shrinkage for the sparsity constrain, are applied sequentially. The algorithm is summarized in Table 1 and Figure 1. Specifically, in Table 1, we use $D_{\tau }\left(X\right)=U\cdot Shrink_{\tau }\left(\Sigma \right)\cdot V^{T}$, where Σ denotes the singular values, following a singular value decomposition of the form $X=U\cdot \Sigma \cdot V^{T}$. Furthermore, we employ $Shrink_{\tau }\left(X\right)=X\cdot \max\left(\left|X\right|-\tau ,0\right)/\left|X\right|$. If, instead of $F_{u}$, we apply the identity matrix I, and instead of *y* the scanner reconstructed image, the above becomes an image decomposition problem.

### 2.3. Sparsifying Transforms

Two of the most common transforms used in CS and RPCA to promote sparsity are wavelets [28,29,30,31,32,33] and temporal Fourier [34]. Wavelets project a signal of finite energy on a frequency subband. This subband is generated by scaling and translating a function $\psi \in L^{2}\left(R^{2}\right)$, the so-called *mother wavelet*, i.e.,

(5) $$\psi_{a,b}=\frac{1}{\sqrt{a}}\psi \left(\frac{t-a}{b}\right).$$

Temporal Fourier is a case of wavelet transform with $e^{2\pi it}$ acting as a mother wavelet. Discontinuities and edges in multidimensional data require a large number of wavelet coefficients to be accurately represented; hence, wavelet representations are not sparse. Shearlets [15,16,18,35,36] utilize the framework of affine systems and are non-isotropic versions of the wavelet transformations that may provide an optimal sparse representation of images. Shearlets, $\psi \in L^{2}\left(R^{2}\right)$, are directional representation systems with composite dilations generated by:

(6) $$\psi_{a,s,b}={\left|M_{a,s}\right|}^{-\frac{1}{2}}\psi \left(M_{a,s}^{-1}\left(t-b\right)\right),\;\mathrm{and}\;M_{a,s}=\left(\begin{array}{cc} a & s\sqrt{a}\\ 0 & \sqrt{a} \end{array}\right),$$

where *s* represents the shear parameter, as in [19]. The shearlet transform of any given image $x\in L^{2}\left(R^{2}\right)$ can be defined as $Sh_{a,s,b}\left(x\right)=\langle x,\psi_{a,s,b}\rangle$. To reduce the computational time, discrete shearlet transforms were computed by the fast Fourier transform, as suggested by Hauser et al. [17].

### 2.4. Image Registration—Motility Metric

Following RPCA reconstruction and decomposition, images were registered with an intensity-based algorithm [10] that minimizes a joint cost function (CF) of the sum of squared differences (SSD) between a reference image $x_{ref}$ and the transformed images $T_{dx,dy}x$, including a transformation model of all intensity changes $c=x_{ref}-T_{dx,dy}x$ and a regularization term *R* that ensures that the deformation fields dx,dy are adequately smooth [10], namely:

(7) CF(dx,dy,c)=SSD(dx,dy,c)+R(dx,dy,c).

The regularization term *R* is based on the second-order spatial derivatives of the displacements dx, dy, and *c*. The chosen algorithm was shown to be able to accurately estimate local displacements that occur during bowel motion.

To assess the motility, as suggested in the corresponding literature [10,11], we employed the Jacobian determinants (*J*) of the displacement fields obtained after registration. In this direction, the motility map, denoted by $\sigma_{J}$, was calculated as the standard deviation over time of the following Jacobian determinant:

(8) $$\sigma_{J}\left(i,j\right)=\sigma \left({\left.J(i,j,t)\right|}_{t=1}^{N_{t}}\right),$$

where *i*, *j* are the spatial coordinates, summed over time *t*. The motility map $\sigma_{J}$ has been validated as a measure of local small bowel motion, and it is claimed to be insensitive to rigid transformation [10,37]. However, respiratory motion is non-rigid and is measured within the motility map. The mean of $\sigma_{J}$, denoted by $\mu (\sigma_{J})$, is used to provide a sense of magnitude of the bowel motility, referred to as the *motility score*. The motility score was calculated from appropriate regions of interest (ROI), drawn around the small bowel [38].

## 3. Materials and Methods

### 3.1. Small Bowel MR Acquisition

Eight subjects were scanned using a Philips Achieva 3T (Philips Healthcare, Eindhoven, The Netherlands) using the manufacturer’s torso coil (XL-TORSO). Subjects were healthy volunteers, 2 males and 6 females, with an age range of 19–42 years and a mean age of 28 years. Patients were imaged using a multi-slice balanced Turbo Field Echo (bTFE) motility sequence, coronal 2.5 × 2.5 × 5 mm${}^{3}$ voxel size, FOV 420 × 420 × 30 mm${}^{3}$, FA = 20 degrees, TE = 1.85 ms, TR = 3.7 ms, with a temporal resolution of 1 volume per second. The first twenty (20) images were acquired on breath-holding; following a 10-s recovery period, the subject was instructed to “gently free-breathe” for a total of 60 images.

### 3.2. Simulated Abdominal DCE Data

A normal volunteer underwent a fast gradient echo DCE-MRI protocol (flip angle 10${}^{\circ }$, repetition time 2.3 ms). T1-weighted images were acquired in multiple time frames without contrast injection. The first time frame was manually segmented into: liver, bowel, right and left heart, aorta, and portal vein. This specific segmentation was used as a map to simulate contrast enhancement, using the extended Tofts model and a population arterial input function. T1 values and pharmacokinetic parameters for each organ were chosen in agreement with a previous study [39]. Fifty DCE images were generated from the ground truth kinetic parameters with temporal resolution of 3 s. More details about the DCE digital phantom can be found in [40].

### 3.3. Simulated Undersampled Acquisition

Original simulated DCE and scanner small bowel images were transformed to (k,t)-space with fast Fourier transformation, where normally distributed noise was added. The level of noise was decided so that the signal-to-noise ratio (SNR) was close to the SNR of DCE. For the undersampling pattern, phase encoding lines were randomly selected per volume and per time frame. The centre of *k*-space was more densely sampled. Cartesian undersampling patterns for 4- and 8-fold acceleration were generated using a Monte Carlo algorithm to generate a sampling pattern with minimum peak interference [12].

### 3.4. Quantitative Evaluation

The techniques described were implemented in MATLAB^®^ R2019b (The Mathworks Inc., Natick, MA, USA). The proposed image processing procedures were conducted on a desktop personal computer with a 2.50 GHz Intel^®^ Core^TM^ i7-4710HQ CPU processor and 16 GB RAM operating memory, running Windows 10 Professional Edition.

The parameter settings for *k*-*t* FOCUSS [22] were selected at 40 inner iterations, 2 outer iterations, weighting matrix power factor 0.5, and initial estimate corresponding to low frequency values. The RPCA reconstruction/decomposition used consistently the following parameter settings: $\lambda_{L}=0.0025$, $\lambda_{S}=0.00125$ for separation of bowel motility from respiration, and $\lambda_{L}=0.01$, $\lambda_{S}=0.01$ for separation of contrast enhancement from background. Similarly to Otazo et al. [6], the regularization parameters with the lowest root mean square error were selected.

### 3.5. Implementation Details

The RPCA reconstruction is evaluated based on the relative reconstruction error, denoted by re, namely:

(9) $$re=\frac{{∥y-y_{e}∥}_{2}^{2}}{{∥y∥}_{2}^{2}},$$

where $y_{e}$ is the estimated (k,t)-space for each method [10]. To evaluate how RPCA could enhance the registration of DCE and small bowel data, deformation fields and Jacobian determinants were estimated by the optical-flow registration algorithm [10]. The *L*, M=L+S of the DCE were registered to derive the deformation fields, and were compared to examine which was closer to the ground truth deformation fields. For the small bowel dataset, a previously validated approach for evaluating the deformation fields was used, namely, the motility score $\mu (\sigma_{J})$ [10]. In this work, motility scores, $\mu (\sigma_{J})$, were derived from the *S* component of the free-breathing data, and were compared to the ones from the breath-holding data. The assumption is that motility scores, $\mu (\sigma_{J})$ estimated from the breath-holding data will be less biased from respiratory motion. Consequently, if the motility scores between the *S* component of the breath-holding and free-breathing data match, then there is a clear indication that we successfully isolated bowel motility in the sparse component.

## 4. Results

### 4.1. Low-Rank Plus Sparse Image Decomposition

**Figure 2:**
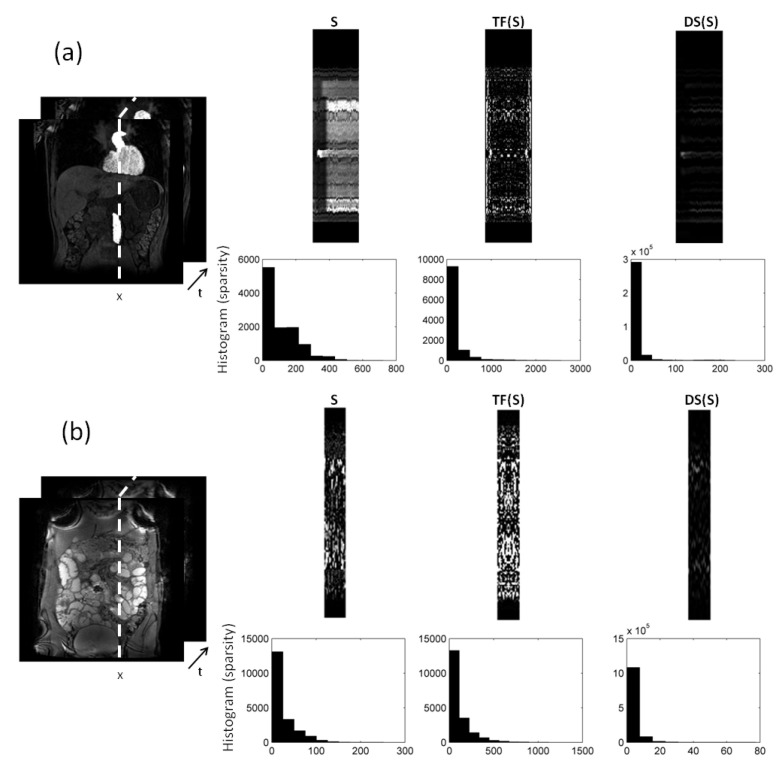
L+S decomposition of: (**a**) 2D abdominal DCE images, and (**b**) 2D small bowel images. Temporal (x−t) profiles along the dotted direction aim to capture the dynamic intensity changes. The low-rank (*L*) in both (**a**) and (**b**) includes the background (and periodic motion), whereas the sparse component captures rapid intensity changes either because of contrast enhancement (**a**), or because of bowel motility (**b**). Increased sparsity can be achieved using Fourier transformation along time (TF) and discrete shearlets. Histograms of the transformed *S* components with TF and DS are shown to illustrate the increased sparsity.

**Figure 3:**
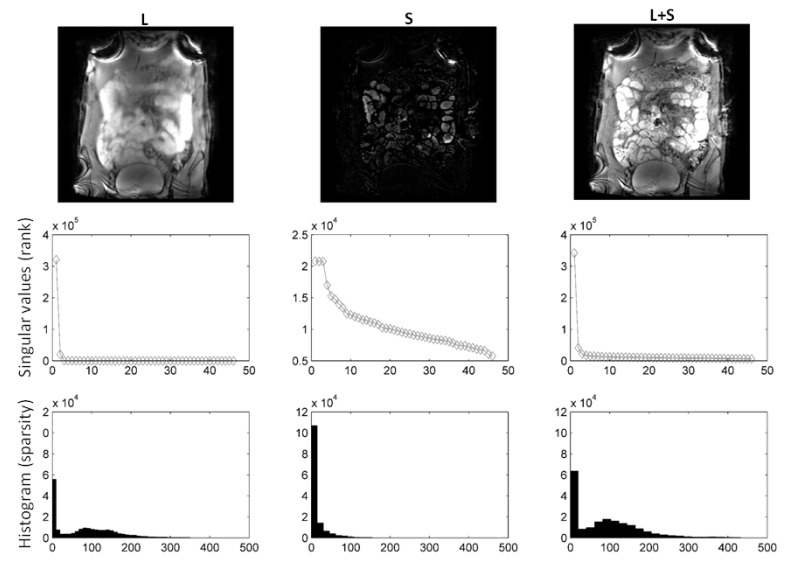
L+S using DS as sparsifying transform decomposition using shearlets as sparsifying transform on small bowel images. Following decomposition, the rank of *L* was 2, whereas the rank of *S* equalled 50. It is clear from the *L*, *S* images and histograms that *S* is sparser than *L*. Consequently, *L* includes background, periodic motion, while *S* captures bowel motility (rapid intensity changes).

**Figure 4:**
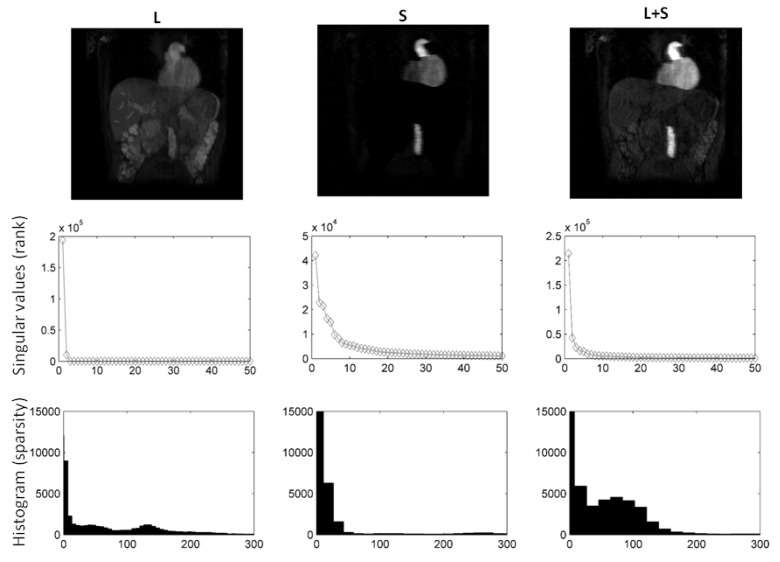
L+S using DS as sparsifying transform decomposition using shearlets as sparsifying transform on abdominal DCE images. Following decomposition, the rank of *L* was 1, whereas *S* is not low-rank. It is clear from the *L* and *S* images and histograms that *S* is far more sparse than *L*. Consequently, *L* includes static background, while *S* captures contrast enhancement (rapid intensity changes).

In Figure 2, we compare different sparsifying transforms, i.e., the unity matrix I, the Fourier transformation along time (TF), and discrete shearlets. Discrete shearlets demonstrated increased sparsity over the other two transforms, for both small bowel and abdominal DCE images. To quantify the increased sparsity achieved with DS, the $L^{0}$-norms of *S*, TF(*S*), and DS(*S*) were estimated. For the abdominal DCE images, the $L^{0}$-norm of *S*, TF(*S*), and DS(*S*) was 1.69 × 10${}^{6}$, 0.88 × 10${}^{6}$, and 0.85 × 10${}^{6}$, respectively; similarly, for the small bowel images, the $L^{0}$-norm was 1.2 × 10${}^{6}$, 0.93 × 10${}^{6}$, and 0.89 × 10${}^{6}$, respectively. The application of L+S using DS as a sparsifying transform in small bowel and abdominal DCE images is shown in Figure 3 and Figure 4, respectively. In both cases, L+S decomposition using DS resulted in low-rank *L* (rank = 2). The sparse component is not low-rank, capturing rapid intensity changes due to bowel motility or contrast enhancement, respectively.

### 4.2. Low-Rank Plus Sparse Image Decomposition from Undersampled (k,t)-Space Data

In Table 2 and Table 3, we compare different reconstruction techniques, namely, *k*-*t* FOCUSS, L+S using the identity matrix, TF, wavelet transform (WT), and DS as sparsifying transforms, in terms of relative reconstruction error (re) of the simulated DCE dataset and of the eight different small bowel datasets for both four- and eight-fold undersampled (k,t)-space data. For the methods presented in Table 2, namely, *k*-*t* FOCUSS, L+S using I, L+S using TF, L+S using WT, and L+S using DS as sparsifying transforms, the average CPU time was 192 s, 450 s, 495 s, 487 s and 1123 s, respectively.

L+S using DS as sparsifying transform showed reduced median re in the simulated DCE dataset and across the eight small bowel datasets compared to the other reconstructions. It is worth mentioning that in Table 3, the asterisk (${}^{*}$) appears when the median values between the L+S reconstructions and *k*-*t* FOCUSS are significantly different. In this direction, we employed the Mann–Whitney U-test, which compared if there was any difference between the independent samples when normality was violated [41]. The Mann–Whitney U-test was utilized in order to evaluate the differences between data samples, and the corresponding *p*-values were calculated.

**Figure 5:**
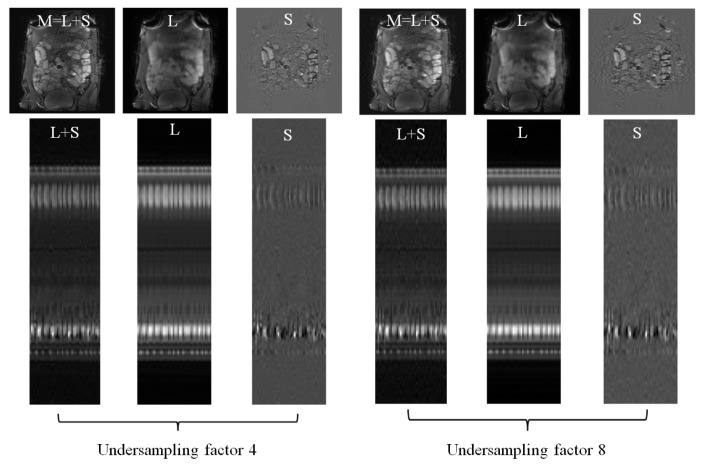
L+S reconstruction using discrete shearlets as sparsifying transforms from small bowel 4-fold and 8-fold undersampled (k,t)-data. *L*, *S*, L+S recovered images, and time-cut representations, as in Figure 2, are illustrated for each undersampling.

Figure 5 illustrates the L+S using DS as sparsifying transform decomposition from four-fold and eight-fold undersampled (k,t)-space data. The magnitudes and the *y*-*t* profiles of the recovered components are presented to qualitatively show that high acceleration factors were achieved while preserving image quality.

### 4.3. Quantification of Motility in Low-Rank Plus Sparse Decomposition Using Discrete Shearlets as Sparsifying Transforms

**Figure 6:**
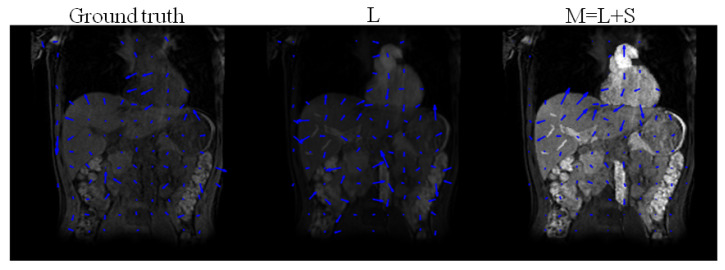
Ground truth deformation fields of the original T1-weighted images (without enhancement) and deformation fields obtained after registration of the low-rank *L*, and M=L+S, following L+S, using DS as sparsifying transform decomposition of the simulated DCE images.

Figure 6 illustrates deformation fields after registering the low-rank (*L*) and the recovered image of the form M=L+S, following the decomposition of the simulated DCE images. Table 4 shows that the relative error between the deformation fields estimated by registering *L* are closer to ground truth deformation fields than the deformation fields estimated by registering M=L+S. Registering *M* assigns intensity changes due to enhancement as deformations, and overestimates the deformations.

**Figure 7:**
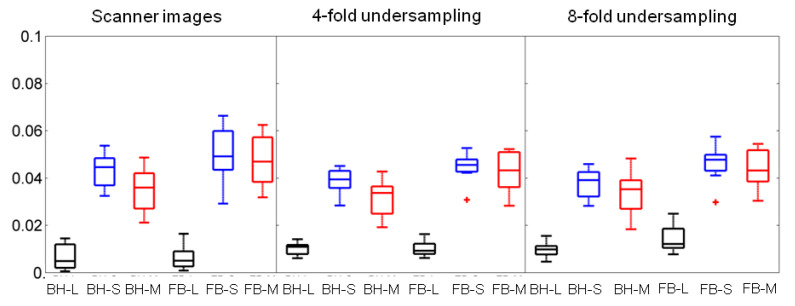
Boxplots of the motility scores across the 8 subjects with range (line), interquartile range (box), and median (horizontal line) for breath-holding (BH) and free-breathing (FB) data. Motility scores were derived with L+S using DS as sparsifying transform decomposition/reconstruction of scanner images/undersampled data. The *L*, *S*, M=L+S components derived from the BH data were compared with the ones from the FB data.

Figure 7 (median, interquartile range) compares, in boxplot format, the motility scores derived from the free-breathing versus the breath-holding data for scanner images, four-fold undersampled (k,t)-space data, and eight-fold undersampled (k,t)-space data, respectively, following L+S using DS as sparsifying transform decomposition/reconstruction. Motility scores were estimated for each component, namely, low-rank *L*, sparse *S*, and low-rank plus sparse M=L+S.

For the scanner images, the median motility scores derived from the sparse component (*S*) were not significantly different between the breath-holding and the free-breathing (median $\mu (\sigma_{J})$ equal to 0.044 and 0.049, respectively, *p* = 0.20). If the median motility scores are calculated from both *L* and *S*, then they are significantly different between the breath-holding and the free-breathing (median $\mu (\sigma_{J})$ equal to 0.036 and 0.047, respectively, *p* = 0.02).

For the four-fold undersampled data, the median motility scores derived from the sparse component (*S*) were not significantly different between the breath-holding and the free-breathing (median $\mu (\sigma_{J})$ equal to 0.039 and 0.045, respectively, *p* = 0.08). If the median motility scores are calculated from both *L* and *S*, then they are significantly different between the breath-holding and the free-breathing (median $\mu (\sigma_{J})$ equal to 0.034 and 0.043, respectively, *p* = 0.02). For the eight-fold undersampled data, the median motility scores derived from the sparse component (*S*) were significantly different between the breath-holding and the free-breathing (median $\mu (\sigma_{J})$ equal to 0.039 and 0.047, respectively, *p* = 0.04). Similarly, when the median motility scores were calculated from both *L* and *S* they were once again significantly different between the breath-holding and the free-breathing (median $\mu (\sigma_{J})$ equal to 0.035 and 0.043, respectively, *p* = 0.01). All calculations described above are summarized in Table 5. To evaluate the effect of undersampling in the value of the motility score, the median motility scores calculated from *L*, *S* and *M* of the free-breathing scanner images were compared to the ones from the four-fold undersampled data (*L*: *p* = 0.1, *S*: *p* = 0.27, *M*: *p* = 0.35) and from the eight-fold undersampled data (*L*: *p* = 0.01, *S*: *p* = 0.45, *M*: *p* = 0.49); for details, see Table 6.

## 5. Discussion

In this paper, we evaluated the benefits of L+S decomposition/reconstruction in deriving accurate deformation fields. RPCA, using discrete shearlets as sparsifying transform, attained less relative reconstruction errors than using other sparsifying transforms (identity matrix or TF) and *k*-*t* FOCUSS, see Table 2 and Table 3. This can be explained given that DS(*S*) was sparser than *S* and TF(*S*) themselves; hence, they were more incoherent with $F_{u}^{T}y$, see Figure 2. L+S using DS as sparsifying transform successfully separated the low-rank (background and periodic motion) from the sparse component (enhancement or bowel motility) for both DCE and small bowel data. This is clearly shown in the histograms and singular values in Figure 3 and Figure 4, and the temporal profiles of Figure 5.

For the simulated DCE dataset, it was shown that deformation fields estimated after registering the low-rank are closer to ground truth than the ones after registering M=L+S. The low-rank plus sparse reconstructed images incorporate the enhancement (sparse component) within themselves; hence, intensity changes due to enhancement are perceived as motility by the registration algorithm.

For the small bowel datasets, following the comparison between breath-holding and free-breathing data (scanner images and four-fold undersampled *k*-space data), it was shown that the motility scores calculated from the sparse component were not significantly different (p>0.05), whereas motility scores calculated from the low-rank plus sparse were significantly different (p<0.05), see Figure 7. This, in essence, means that when motility scores are calculated from the sparse component (that excludes low-rank periodic motion), the breath-holding motility scores are similar to the free-breathing ones. Furthermore, in terms of the effect of undersampling in the calculation of the motility scores, there were no significant differences between the ones calculated from the scanner images and the four-/eight-fold undersampled data.

This work suggests the use of discrete shearlets as the sparsifying transform of choice for low-rank plus sparse reconstruction/decomposition. Discrete shearlets accurately represent the discontinuities and edges occurring in MR imaging and, hence, will be sparser than other transforms. RPCA reconstructions are expected to reduce the bias due to undersampling, and to separate small bowel motility from respiration. The improved reconstruction performance of the proposed L+S using DS as sparsifying transform over other undersampling schemes, such as *k*-*t* FOCUSS, will allow us to either improve the temporal resolution or keep the same temporal resolution and improve the spatial resolution, thus separating the respiratory motion, corresponding to the low-rank component, from the enhancement or bowel motility, corresponding to the sparse component.

## 6. Conclusions

The choice of sparsifying transform in an RPCA decomposition/reconstruction algorithm is important to reconstruct unbiased images from undersampled data. Discrete shearlets provide an excellent representation of multi-dimensional MR data. In this paper, we utilized discrete shearlets as sparsifying transforms in low-rank rank plus sparse decomposition/reconstruction. In this direction, discrete shearlets were shown to reduce relative reconstruction errors, compared to other sparsifying transforms. Low-rank plus sparse decomposition using discrete shearlets was evaluated in DCE and small bowel data. Registering low-rank reconstructed DCE images accurately quantifies respiratory motion. Motility scores of small bowel images are not significantly affected by undersampling. When estimated from the sparse components, motility scores are not significantly different between the breath-holding and free-breathing data, thus indicating that the proposed algorithm was able to isolate respiratory motion in the low-rank component. In future studies, we intend to include learned priors and sparsifying operators, as well as machine-learning methods in the low-rank plus sparse decomposition approach. This study focused on dynamic 2D MRI acquisitions; hence, only 2D shearlets were applicable. However, we expect that, for 3D MRI acquisitions, the benefit of 3D shearlets would be more significant, compared to other 3D transformations, such as wavelets and Fourier.

## Figures and Tables

**Table 1:** Synopsis of L+S decomposition and reconstruction algorithm, as in [6].

L+Φ(S) decomposition and reconstruction algorithm
**Input**: (k,t)-space samples y, decomposition parameters $\lambda_{L}$, $\lambda_{S}$
**Initialize**: $M^{1}=F_{u}^{T}y$ and $S^{1}=0$, iteration *k*=1;
**while** stopping criterion is not met, do
Singular value thresholding
$L^{k+1}=D_{\lambda_{L}}\left(M^{k}-S^{k}\right)$
Shrinkage operator
$S^{k+1}=\Phi^{-1}\left(Shrink_{\lambda_{S}}\left(\Phi \left(M^{k}-L^{k}\right)\right)\right)$
Subtract aliasing artifacts from M=L+S
$M^{k+1}=L^{k+1}+S^{k+1}-F_{u}^{T}\left(F_{u}\left(L^{k+1}+S^{k+1}\right)-y\right)$
Stopping criterion
k>50 or $\frac{{∥M^{k+1}-M^{k}∥}_{2}}{{∥M^{k}∥}_{2}}<10^{-5}$
**end while**
**Output**: $L=L^{k+1}$, $S=S^{k+1}$

**Table 2:** Relative reconstruction error (re) of the L+S reconstructions, using the identity matrix I, TF, WT, and DS as sparsifying transforms and *k*-*t* FOCUSS for the simulated DCE datasets.

	Unders. Factor 4	Unders. Factor 8
*k*-*t* FOCUSS	0.162	0.203
L+S using I as sparsifying transform	0.149	0.194
L+S using TF as sparsifying transform	0.147	0.189
L+S using WT as sparsifying transform	0.147	0.188
L+S using DS as sparsifying transform	0.144	0.187

**Table 3:** Median and interquartile range (iQR) of relative reconstruction error (re) of the reconstructions *k*-*t* FOCUSS, L+S reconstructions (using identity matrix I, TF, WT, and DS as sparsifying transforms), across the 8 small bowel datasets.

**Undersampling Factor 4**	**Median**	**iQR**
*k*-*t* FOCUSS	0.077	0.013
L+S using I as sparsifying transform	0.080	0.012
L+S using TF as sparsifying transform	0.079	0.012
L+S using WT as sparsifying transform	0.075	0.011
L+S using DS as sparsifying transform	0.064 *	0.006
**Undersampling Factor 8**	**Median**	**iQR**
*k*-*t* FOCUSS	0.120	0.025
L+S using I as sparsifying transform	0.119	0.018
L+S using TF as sparsifying transform	0.115	0.017
L+S using WT as sparsifying transform	0.112	0.021
L+S using DS as sparsifying transform	0.106	0.029

**Table 4:** Relative error between the ground truth deformation fields and the ones obtained after registration of the low-rank (*L*), and M=L+S following L+S using DS as sparsifying transform decomposition of the simulated DCE images and reconstruction from the 4/8-fold undersampled DCE (k,t)-space data (US4/US8).

	*L*	M=L+S
DCE images	0.016	0.031
US4	0.021	0.025
US8	0.024	0.029

**Table 5:** Median motility scores $\mu (\sigma_{J})$ for *S* and L+S in the breath-holding and free-breathing data.

Scanner images	BH	FB	*p*-value
*S*	0.044	0.049	0.20
L+S	0.036	0.047	0.02
**Four-fold**	BH	FB	*p*-value
*S*	0.039	0.045	0.08
L+S	0.034	0.043	0.02
**Eight-fold**	BH	FB	*p*-value
*S*	0.039	0.047	0.04
L+S	0.035	0.043	0.01

**Table 6:** *p*-values measuring the effect of undersampling in the value of the motility score: median motility scores calculated from *L*, *S*, and *M* of the free-breathing scanner images, compared to the ones from the four-fold and eight-fold undersampled data, respectively.

	*L*	*S*	M=L+S
Four-fold	0.10	0.27	0.35
Eight-fold	0.01	0.45	0.49

## Data Availability

The simulated data presented in this study are available on request from the corresponding author.
